# Effectiveness of mHealth diet interventions in cancer survivors: A systematic review and meta-analysis of randomized controlled trials

**DOI:** 10.1016/j.apjon.2023.100196

**Published:** 2023-02-14

**Authors:** Yabo Gong, Xiaohan Jiang, Xijie Chen, Shi Chen, Yuee Wen, Xiuhong Yuan, Jiamin Chen, Junsheng Peng

**Affiliations:** a School of Nursing, Sun Yat-sen University, Guangzhou, China; b The Sixth Affiliated Hospital, Sun Yat-sen University, Guangzhou, China; c Guangdong Institute of Gastroenterology, Guangdong Provincial Key Laboratory of Colorectal and Pelvic Floor Diseases, Supported by National Key Clinical Discipline, Guangzhou, China; d State Key Laboratory of Oncology in South China, Collaborative Innovation Center for Cancer Medicine, Sun Yat-Sen University Cancer Center, Guangzhou, China; e Department of Clinical Nutrition, the Sixth Affiliated Hospital, Sun Yat-sen University, Guangzhou, China

**Keywords:** mHealth, Cancer survivors, Diet, Systematic review, Meta-analysis

## Abstract

**Objective:**

To evaluate the effects of mobile health (mHealth) diet interventions on cancer survivors’ diet intake, weight change, waist circumference, hip circumference, and quality of life (QoL).

**Methods:**

The PubMed, Embase, Web of Science, Cochrane Library, Scopus, ProQuest, China National Knowledge Infrastructure, Wanfang, and SinoMed databases were searched from their inception to September 25, 2022. Randomized controlled trials (RCTs) on the effects of mHealth diet interventions in cancer survivors were identified. Two researchers independently selected the included studies and appraised their quality. The methodological quality of the included studies was assessed using the Revised Cochrane risk-of-bias tool for RCTs (RoB2).

**Results:**

A total of 15 RCTs involving 2363 cancer survivors were included. MHealth diet interventions significantly improved fruit and vegetable intake (standardized mean difference [SMD] ​= ​0.19, 95% confidence interval [CI] [0.05, 0.33], *P* ​< ​0.01), and QoL (SMD ​= ​0.13, 95% CI [0.01, 0.26], *P* ​= ​0.04) and reduced fat intake (SMD ​= ​−0.22, 95% CI [−0.34, −0.11], *P* ​< ​0.01), weight (SMD ​= ​−0.35, 95% CI [−0.48, −0.22], *P* ​< ​0.01), waist circumference (MD ​= ​−1.43, 95% CI [−2.33, −0.53], *P* ​< ​0.01), and hip circumference (MD ​= ​−3.54, 95% CI [−4.88, −2.19], *P* < 0.01) in cancer survivors. No significant differences were observed in energy intake (*P* ​= ​0.46) or whole grain intake (*P* ​= ​0.14).

**Conclusions:**

MHealth diet interventions may be an effective strategy for cancer survivors. Large-scale RCTs with rigorous study designs are needed to examine the effect of diet intervention delivered via mHealth.

## 1 Introduction

In recent years, the number of cancer survivors has substantially grown.^1^ Early detection of cancer and improvements in treatments have enhanced survival rates for cancer survivors.^2^ However, the diagnosis and treatment of cancer impose physical and psychological burdens on cancer survivors.^3^^,^^4^ In particular, physiological changes associated with the tumor (such as malabsorption, obstruction, and diarrhea), the host response to the tumor (anorexia and altered metabolism), and the side effects of cancer treatment (nausea and loss of appetite) can lead to poor nutritional status in cancer survivors^5^, ^6^, ^7^. Research indicates that a healthy diet is associated with better clinical outcomes, quality of life (QoL), and overall survival in cancer survivors^8^, ^9^, ^10^. The goals of diet interventions include improving nutritional status, achieving and maintaining a healthy weight, and improving the QoL of cancer survivors.^11^^,^^12^ Hence, it is important to provide effective diet interventions to cancer survivors to improve their prognosis.

Mobile health (mHealth) is defined as a “medical and public health practice supported by mobile devices”^13^ and is widely used for diet interventions in cancer survivors. MHealth is delivered through various tools, including phone calls, text messages, websites, and applications to provide tailored information, frequent interactions, and high-quality medical services.^14^^,^^15^ MHealth thus provides increased ubiquity of interventions, with the ability to reach users at nearly any time or place to deliver timely feedback.^16^ Studies have shown that mHealth diet interventions can change patients' health behavior and may be efficacious in cancer survivors.^17^^,^^18^

There is considerable debate regarding the value of mHealth nutritional interventions in cancer survivors. Most of the previous studies that evaluated the effectiveness of mHealth diet interventions in cancer survivors and evaluated a positive effect^19^^,^^20^; however, others studies did not.^21^^,^^22^ Thus, mHealth dietary interventions have not been conclusively shown to improve the diet intake of cancer survivors, and it remains unclear which intervention components are effective.

Previous systematic reviews focused on the ability of mHealth interventions to increase physical activity or improve psychological symptoms in cancer survivors.^23^^,^^24^ Another systematic narrative review evaluated electronic health interventions delivered to cancer survivors as well as changes in diet behavior and QoL, but they did not conduct a quantitative meta-analysis.^25^ The effect of mHealth diet interventions on diet intake, body composition, and QoL has not been examined. Therefore, this meta-analysis aimed to (1) use available evidence to quantitatively determine the effects of mHealth diet interventions, including diet intake, weight change, waist circumference, hip circumference, and QoL, in cancer survivors, and (2) provide a scientific basis for the clinical application of mHealth diet interventions in cancer survivors.

## 2 Methods

The methods adhered to the Preferred Reporting Items for Systematic Reviews and Meta-Analyses (PRISMA) statement.^26^

### 2.1 Inclusion and exclusion criteria

#### 2.1.1 Inclusion criteria

Studies that met the following criteria were included: (1) Population: Patients who were cancer survivors (any type or stage) and aged above 18 years. (2) Intervention: mHealth diet interventions using phone calls, text messages, websites, or mobile applications to improve cancer survivors’ diet intake. (3) Control groups that received the usual care, wait-list, and printed materials. (4) Outcomes: The outcome includes diet intake, weight change, waist circumference, hip circumference, or QoL. (5) Study: The research design was a randomized controlled trial (RCT).

#### 2.1.2 Exclusion criteria

We excluded studies that (1) were research protocols, conference abstracts, comments, or case reports; (2) provided mHealth interventions (sending messages through mHealth technology, such as phone, emails, or newsletters) to the control group; (3) without adequate data for outcome analysis; or (4) were published in languages other than English or Chinese.

### 2.2 Search strategy

A comprehensive search of the literature was performed in nine electronic databases, including PubMed, Embase, Web of Science, Cochrane Library, Scopus, ProQuest, China National Knowledge Infrastructure, Wanfang, and SinoMed databases from inception until September 25, 2022. We also reviewed the references of the selected publications. The search strategies involved a combination of Medical Subject Headings (MeSH) terms and free text terms. The search strategy was as follows: (neoplasm OR tumor OR cancer) AND (mHealth OR mobile applications OR telephone) AND (diet OR diets OR dietary intake) AND (randomized controlled trial OR randomised controlled trial). The list of search strategies for the nine databases are presented in Supplementary Table S1.

### 2.3 Screening and data extraction

All records were imported to Endnote X9, and duplicates were removed. Two reviewers (GYB and JXH) independently screened the titles and abstracts of identified records. The full texts of selected publications were assessed in detail according to the inclusion criteria by two independent reviewers (GYB and JXH) in the Endnote X9 library. Full texts that did not meet the inclusion criteria were excluded. In cases of disagreement, a third reviewer was consulted (PJS), and a consensus was reached through discussion. If data on the outcome were missing, we contacted the authors by email to request these data.

Researchers extracted data using Microsoft Excel software. The extracted details included the following: (1) general information (eg, the first author's name, publication year, study design, and the country where the research was carried out); (2) characteristics of the cancer patient population (eg, participant demographic characteristics, diagnoses, and sample size); (3) intervention characteristics (eg, intervention type, interveners, mHealth diet intervention, frequency, duration, and details); and (4) outcome indicators and measurement tools.

### 2.4 Quality assessment

Risk of bias of the included RCTs was assessed by two reviewers (GYB and JXH) using the Revised Cochrane risk-of-bias tool for randomized trials (RoB2).^27^ Five domains were evaluated, including the risk of bias from the randomization process, deviations from intended interventions, missing outcome data, measurement of the outcome, and selection of the reported result. Bias in selection of the reported result is a type of reporting bias. Each study was classified in each domain was divided into low risk of bias, high risk of bias or some concerns (Fig. 2). When there were disagreements, the third reviewer (PJS) was consulted, and a consensus was reached through discussion.

### 2.5 Data analysis

The meta-analysis was conducted using RevMan 5.4.1 software. Stata version 17.0 was used to perform funnel chart analyses and Egger's regression test. *P* ​< ​0.05 was taken to indicate significant publication bias.^28^ Standardized mean differences (SMDs) or mean differences (MDs) were calculated. MDs were used to calculate results obtained using the same measurement method. If different scales were used to evaluate the same outcome, SMDs were used. The effect size was calculated using the 95% confidence interval (CI). The heterogeneity was determined by the *Q* test and *I*^*2*^ statistics. In addition, for homogeneous data sets, *P* ​> ​0.1 and *I*^*2*^ < 50% were used as the test thresholds. When these two statistical conditions were met, a fixed-effects model was used for the meta-analysis because the pooled effect sizes were relatively homogenous. If one of the above conditions was not met, a random-effects model and sensitivity analysis were applied to control heterogeneity.^29^^,^^30^ *P* ​< ​0.05 was considered statistically significant.

## 3 Results

### 3.1 Study selection

**Fig. 1:**
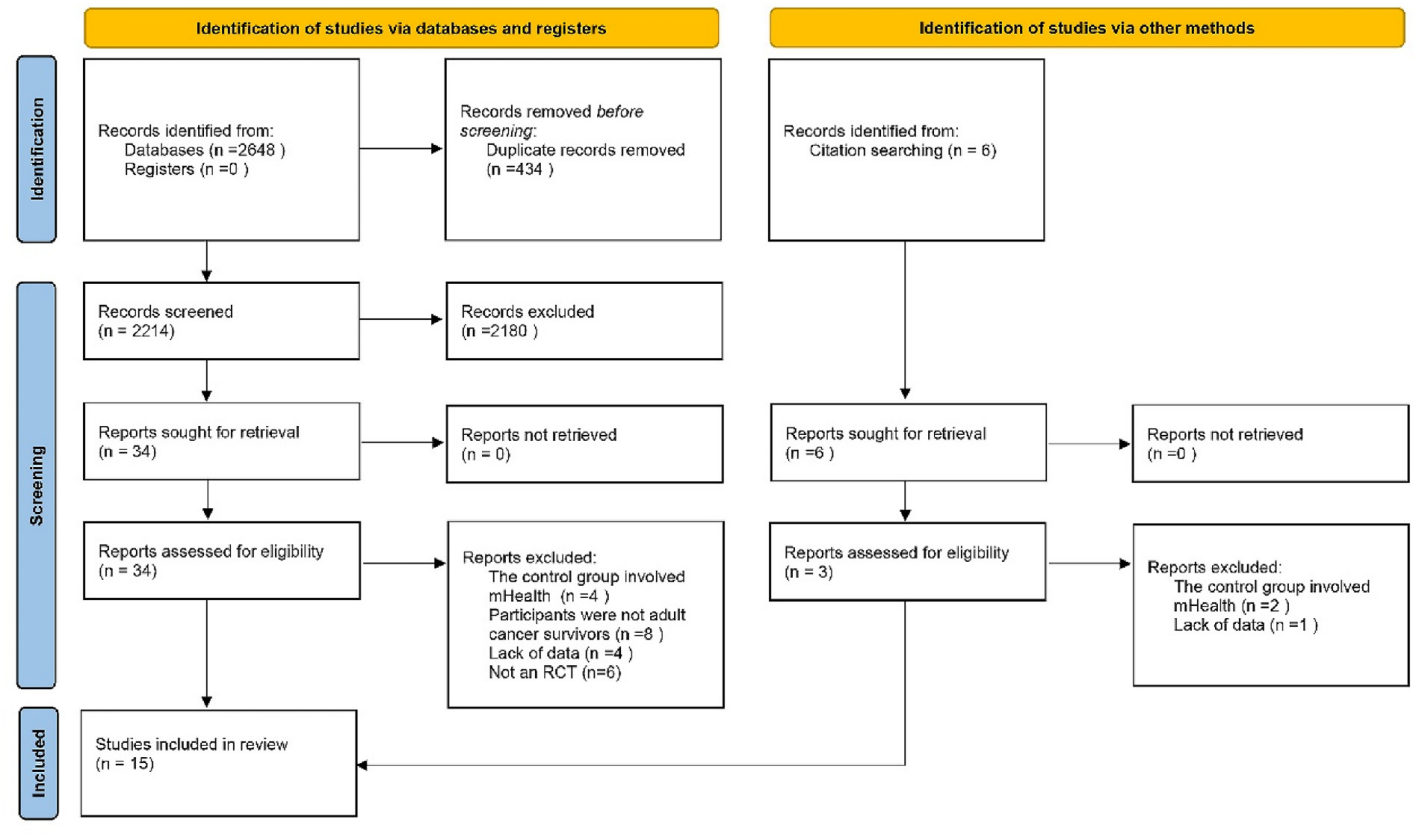
Literature screening flow chart.

As shown in Fig. 1, a total of 2648 articles were initially retrieved. After duplicates were removed, a total of 2214 articles were screened at different stages. First, 2180 articles were removed after by reviewing their titles and abstracts; and further reviews were performed of the full texts of 34 articles. A total of 22 studies were excluded after full-text screening, and the main reasons for exclusion are shown in Fig. 1. In addition, we identified six relevant cited references and finally included three additional studies. In total, 15 articles were included in this study.

**Fig. 2:**
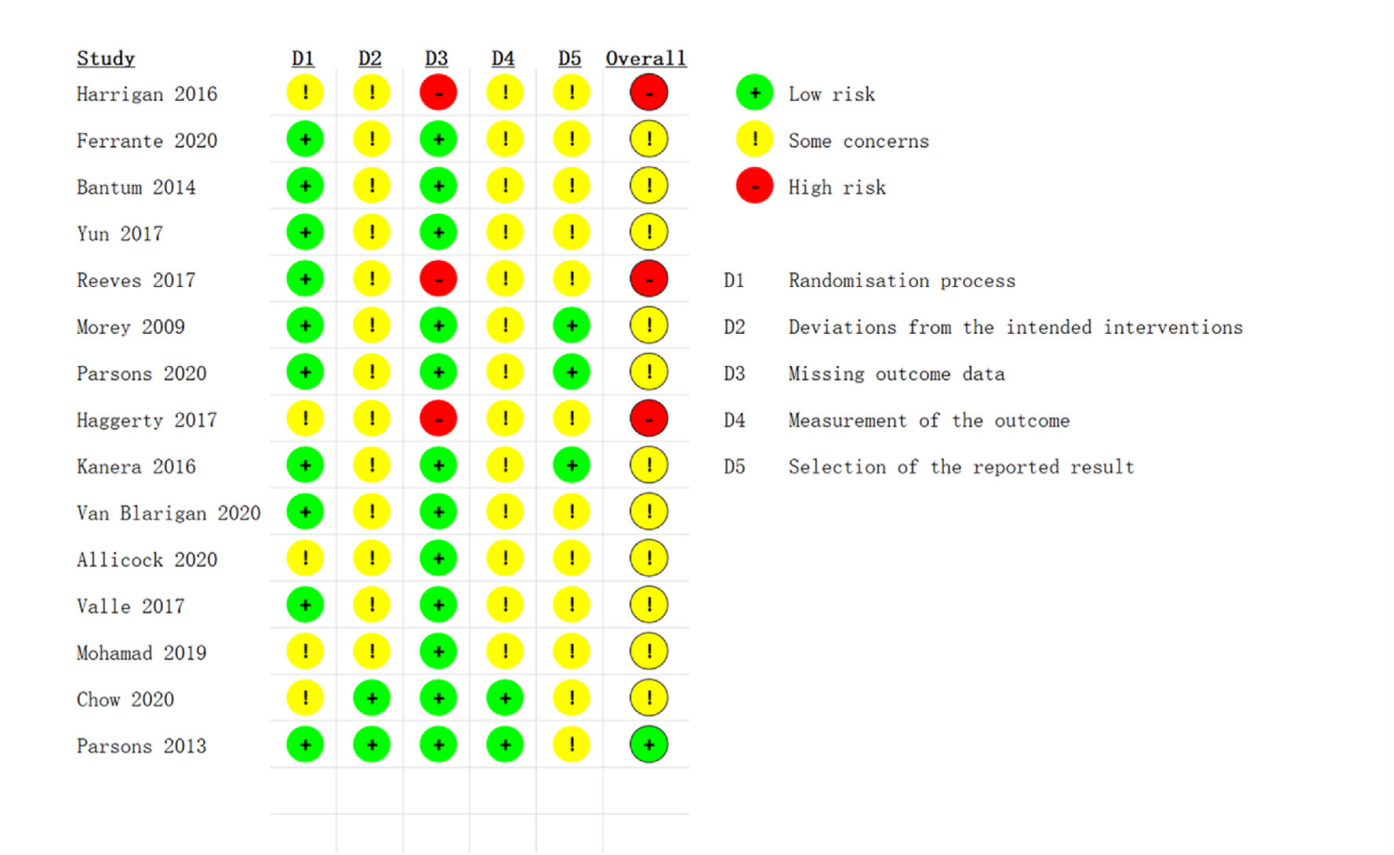
Risk of bias for the included randomized controlled trials.

### 3.2 Study characteristics

After screening, a total of 15 studies involving 2363 participants were included (Table 1). All included articles were published between May 2009 and April 2021. These studies mainly involved patients with breast cancer, prostate cancer, or bladder cancer. The locations of the studies were the United States (10 studies), Australia (two studies), the Netherlands (one study), the United Kingdom (one study), and South Korea (one study). The mean age (standard deviation) was 61.24 years (17.08). The sample size of the studies ranged from 21 to 641 participants.

**Table 1:** Characteristics of the included studies.

Author, Year, Country	Study design	Sample size (IG/CG)	Population, Mean age (SD)	Intervention types for IG	Intervention types for CG	Interveners	MHealth diet intervention	Intervention frequency	Intervention duration	Outcomes	Instrument
Harrigan et al. (2016) Australia^19^	RCT	34/33	Breast cancer, 59 (7.5)	Phone calls	Usual care	Dietitian	Individualized diet counseling (decrease energy intake and plant-based diet)	Participants received individualized counseling sessions once per week (month 1), then every two weeks (months 2 and 3), and once per month (months 4, 5, and 6)	6 months	Fat intake Fruit and vegetable intake Weight Waist circumference Hip circumference	1. 120-item food frequency questionnaire 2. Stadiometer
Ferrante et al. (2020) US^20^	RCT	18/17	Breast cancer, 61.54 (8.83)	Website	Wait-list	–	Education on goals for caloric intake (1200–1500 ​kcal daily)	At least weekly	6 months	Energy intake Weight Waist circumference QoL	1. Sparkpeople.com food diary tool 2. calibrated digital scale 3. Tape 4. QLACS
Bantum et al. (2014) US^21^	RCT	156/147	Cancer, 50.85 (11.09)	Website	Wait-list	Facilitators who were cancer survivors	Educational sessions (such as, improving diet by making healthier food choices)	Weekly	6 months	Fruit and vegetable intake	Block Food Frequency Questionnaire
Yun et al. (2017) South Korea^22^	RCT	92/50	Cancer, 50.68 (9.43)	Phone calls	Print materials	Health master coaches were health professionals Health partners were long-term cancer survivors	Individual coaching (such as balanced diet)	A total of 16 sessions of tele-coaching were conducted: 30 ​min per week for 12 sessions, 30 ​min per 2 weeks for 2 sessions, and 30 ​min per month for 2 sessions were offered for the intervention group.	3 months	QoL	EORTC QLQ-C30, 2. Questionnaire based on the " Rules for National Cancer Prevention: Dietary Practice Guideline"
Reeves et al. (2017) Australia^31^	RCT	40/34	Breast cancer, 55.3 (8.7)	Phone calls	Usual care	Dietitians	Individualized guidance to achieve a kilojoule goal	Telephone calls: weekly for 6 weeks followed by 10 fortnightly calls	6 months	Weight Waist circumference Hip circumference QoL	1. Calibrated scales 2.tape 3.SF-36 Version 2 Health Survey
Morey et al. (2009) US^32^	RCT	319/322	Cancer, 73.05 (5.05)	Phone calls	Wait-list	–	Counseling on healthy caloric-restricted diet	15 sessions and eight prompts over the 12-month period	12 months	Fat intake Fruit and vegetable intake Weight QoL	1. Interactive NDSR software 2.SF-36
Parsons et al. (2020) US^33^	RCT	226/217	Prostate cancer, 63.6 (6.54)	Phone calls	Print materials	–	Counseling on increase daily fruit and vegetable servings	The first phase: 6 counseling telephone calls over 1 month; the second: 4 calls over 2 months; the third: 4 calls over 4 months; and the fourth: 8 calls over 16 months	24 months	Energy intake Fat intake Whole grain intake	NDS-R (version 2010, University of Minnesota Nutrition Coordinating Center, University of Minnesota, Minneapolis).
Haggerty et al. (2017) US^34^	RCT	11/10	Endometrial cancer, 59.7 (8.7)	Phone calls	Usual care	Doctoral students in clinical psychology and medical students	Counseling on tracking caloric intake	1–16 ​Weeks: weekly; 18–24 ​Weeks: biweekly	6 months	Weight QoL	1. conventional scale 2. SF-12
Kanera et al. (2016) Netherlands^32^	RCT	184/219	Cancer, 55.9 (11.39)	Website	Usual care	Fully automated expert system without human involvement.	Education on increase healthy eating behaviors through fruit, vegetable, whole grain, and fish consumption	–	6 months	Fruit and vegetable intake	8 items of the Dutch Standard Questionnaire on Food Consumption
Van Blarigan et al. (2020) US^35^	RCT	22/23	Colorectal cancer, 55 (4.44)	Website	Print materials	–	Education on increase vegetables, whole grain, and fish and decrease processed meats, sugar-sweetened beverages, and alcohol	Everyday	12 weeks	Whole grain intake	ASA24
Allicock et al. (2020) US^36^	RCT	13/9	Breast cancer, 52.23 (9.21)	Mobile app	Wait-list	–	Education on eating more healthfully and managing weight	Everyday	4 weeks	Fruit and vegetable intake Weight Waist circumference	The National Health Interview Survey 2000
Valle et al. (2017) US^37^	RCT	13/11	Breast cancer, 53.21 (8.93)	Emails	Wait-list	Doctor in nutrition	Education on change in eating behavior (decrease energy intake)	24 weekly emails	6 months	Energy intake Weight Waist circumference	1. ASA-24 2.calibrated digital scale 3. Gulick II tape
Mohamad et al. (2019) UK^38^	RCT	26/28	Prostate cancer, 65.5 (5.6)	Phone calls; Website	Wait-list	Dietitian	Counseling on energy reduction (through decreasing portion sizes, reducing high-energy, high-fat, high-sugar foods, reducing alcohol and encouraging higher consumption of fruits, vegetables and wholegrain)	Three consultations at 4-week intervals	12 weeks	Weight QoL	1.calibrated digital scales 2. EORTC QLQ-C30
Chow et al. (2020) US^39^	RCT	24/17	Hematologic malignancies, 40.03 (26.58)	Text messages; Emails; Facebook	Usual care	–	Education on reduce three dietary components (sodium, saturated fats, and added sugars) intake	–	16 weeks	QoL	PROMIS Global 10
Parsons et al. (2013) US^40^	RCT	30/18	Bladder cancer, 66 (62.35)	Phone calls; Skype calls	Print materials	Trained counselors	Education on diet behavior (7 daily vegetable servings, with at least 2 of these as cruciferous vegetables)	5 calls during month 1 and 3 calls during month 2, followed by monthly maintenance calls during months 3–6.	6 months	Energy intake Fat intake Fruit and vegetable intake Whole grain intake	3 separate 24-h dietary recalls

IG, intervention group; CG, control group; RCT, randomized controlled trial; QoL, quality of life; QLACS, Quality of Life in Adult Cancer Survivors Scale; EORTC QLQ-C30, European Organization for Research and Treatment of Cancer Quality of Life Questionnaire; SF-36, Short Form-36; NDSR, Nutrition Data System for Research; NDS-R, Nutrition Data System for Research software and nutrient database; SF-12, 12-Item Short Form Health Survey; ASA24, National Cancer Institute's Automated Self-administered Dietary Assessment Tool; ASA-24, Automated Self-Administered 24-Hour Dietary Recall; PROMIS Global 10, Patient-Reported Outcomes Measurement Information System.

All of the included studies compared mHealth interventions with usual care, wait-lists, or printed materials. Six studies delivered mHealth interventions by phone calls^19^^,^^22^^,^^31^^,^^33^^,^^34^^,^^41^, four studies by a website,^20^^,^^21^^,^^32^^,^^35^ one study by a mobile app,^36^ one study by emails,^37^ one study by a combination of phone calls and a website,^38^ one study by a combination of phone calls and Skype calls,^42^ and another study by a combination of text messages, emails, and Facebook.^39^ Nearly half of the interventionists were dietitians,^19^^,^^31^^,^^38^ cancer survivors,^21^ doctors in nutrition,^37^ trained counselors,^40^ health professionals and cancer survivors,^22^ doctoral students in clinical psychology and medical students,^41^ or fully automated expert systems without human involvement.^32^ Interventions included healthier food choices, decreased energy intake, and reduced dietary component intake. The length of the interventions of the included studies ranged from 4 weeks to 24 months. The frequency of the interventions ranged from daily to 22 times over 24 months. The most common frequency and length of interventions were daily and 6 months, respectively.

### 3.3 Assessing risk of bias

The RoB2 was used to assess the risk of bias in RCTs. Four studies stated that random assignment was performed but did not elaborate at length. We did not obtain adequate information about the blinding procedures for participants, interventionists, or assessors form 13 studies. In the remaining studies, one study blinded outcome assessors, and another blinded outcome assessors and interventionists, but participants were not blinded to their group allocation. Three studies were identified as having a potential risk of bias because of missing outcome data. Twelve studies have some concerns due to missing protocols. In our study, one study was categorized as having a low risk of bias, 11 studies had some risk of bias, and three studies had a high risk of bias (Fig. 2).

### 3.4 Effects of interventions

#### 3.4.1 Effects of mHealth diet interventions on energy intake

**Fig. 3:**
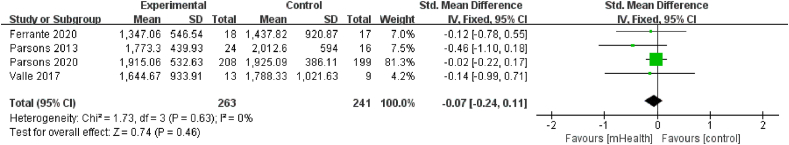
Effects of mHealth diet interventions on energy intake.

Energy intake was measured in four trials^20^^,^^34^^,^^37^^,^^40^ involving 504 patients using the Sparkpeople.com food diary tool, 3 separate 24-hour dietary recalls, Nutrition Data System for Research software and nutrient database (NDS-R), and Automated Self-Administered 24-Hour Dietary Recall (ASA-24). There was no significant heterogeneity among studies (*I*^*2*^ ​= ​0%, *P* ​= ​0.63); thus, the fixed-effects model was used. MHealth diet interventions had no significant effect on energy intake compared with the control conditions (SMD ​= ​−0.07, 95% CI [−0.24, 0.11], *P* ​= ​0.46; Fig. 3).

#### 3.4.2 Effects of mHealth diet interventions on fat intake

**Fig. 4:**
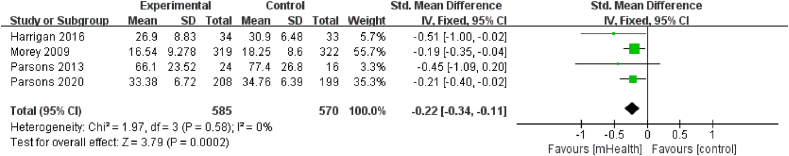
Effects of mHealth diet interventions on fat intake.

Four trials^19^^,^^33^^,^^34^^,^^40^ with 1155 participants assessed the effects of mHealth diet interventions on cancer survivors’ fat intake by using 120-item food frequency questionnaire, interactive Nutrition Data System for Research (NDSR) software, 3 separate 24-hour dietary recalls, and NDS-R. In a fixed-effects model, the analysis revealed a significant effect of mHealth diet interventions on fat intake (SMD ​= ​−0.22, 95% CI [−0.34, −0.11], *P*＜0.01) with no substantial heterogeneity (*I*^*2*^ ​= ​0%, *P* ​= ​0.58; Fig. 4).

#### 3.4.3 Effects of mHealth diet interventions on fruit and vegetable intake

**Fig. 5:**
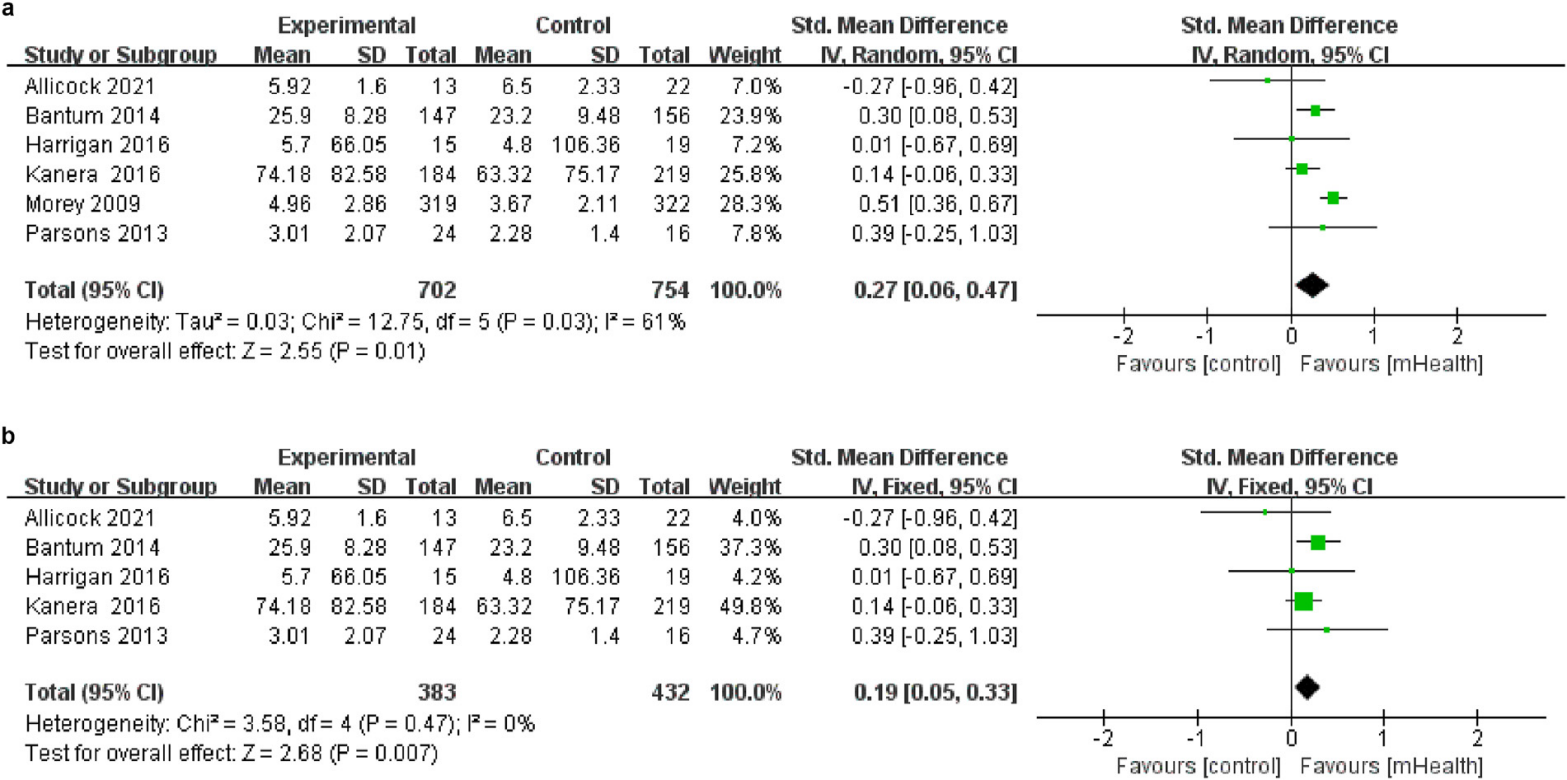
(a) Effects of mHealth diet interventions on fruit and vegetable intake. (b) Sensitivity analysis of mHealth diet interventions on fruit and vegetable intake.

Six studies,^19^^,^^21^^,^^32^^,^^33^^,^^36^^,^^38^ involving 1456 patients evaluated fruit and vegetable intake. Fruit and vegetable intake was measured by using 120-item food frequency questionnaire, Block Food Frequency Questionnaire, 8 items of the Dutch Standard Questionnaire on Food Consumption, interactive NDSR software, The National Health Interview Survey 2000, 3 separate 24-h dietary recalls. A random effect model was used because of high heterogeneity (*I*^*2*^ ​= ​61%, *P* ​= ​0.03; Fig. 5a). To reduce this significant heterogeneity, a sensitivity analysis was conducted by sequentially excluding individual papers. After one^33^ study was removed, no significant heterogeneity was observed among the remaining five studies (*I*^*2*^ ​= ​0%, *P* ​= ​0.47; Fig. 5b). The fixed-effects model showed that there was a positive effect of mHealth diet interventions on fruit and vegetable intake (SMD ​= ​0.19, 95% CI [0.05, 0.33], *P* ​< ​0.01; Fig. 5b).

#### 3.4.4 Effects of mHealth diet interventions on whole grain intake

**Fig. 6:**
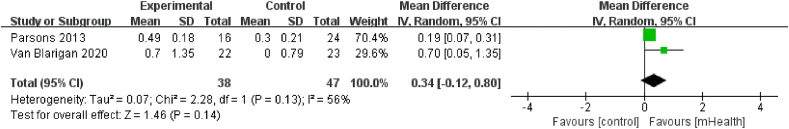
Effects of mHealth diet interventions on whole grain intake.

Two studies,^35^^,^^40^ involving 85 patients, evaluated whole grain intake. Whole grain intake was measured by using National Cancer Institute's Automated Self-administered Dietary Assessment Tool (ASA24) and NDS-R. The results showed that mHealth diet interventions had no significant effect on whole grain intake compared with the control conditions (MD ​= ​0.34, 95% CI [−0.12, 0.80], *P* ​= ​0.14; Fig. 6). A random effect model was used because of the high heterogeneity (*I*^*2*^ ​= ​56%, *P* ​= ​0.13; Fig. 6).

#### 3.4.5 Effects of mHealth diet interventions on weight change

**Fig. 7:**
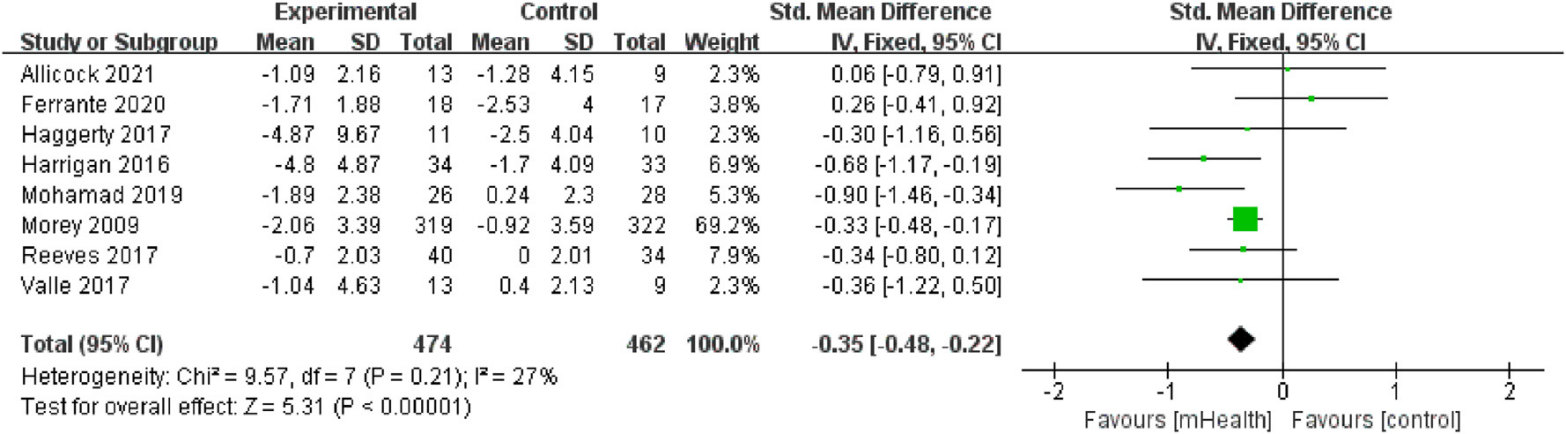
Effects of mHealth diet interventions on weight change.

Eight studies^19^^,^^20^^,^^31^^,^^33^^,^^36^, ^37^, ^38^^,^^41^, involving 936 patients, evaluated weight change. Weight was measured by using stadiometer, calibrated scales, and calibrated digital scale. There was no significant heterogeneity among studies (*I*^*2*^ ​= ​27%, *P* ​= ​0.21); thus, a fixed-effects model was used. MHealth diet interventions had a statistically significant effect on weight loss compared with the control conditions (SMD ​= ​−0.35, 95% CI [−0.48, −0.22], *P* ​< ​0.01; Fig. 7).

#### 3.4.6 Effects of mHealth diet interventions on waist circumference

**Fig. 8:**
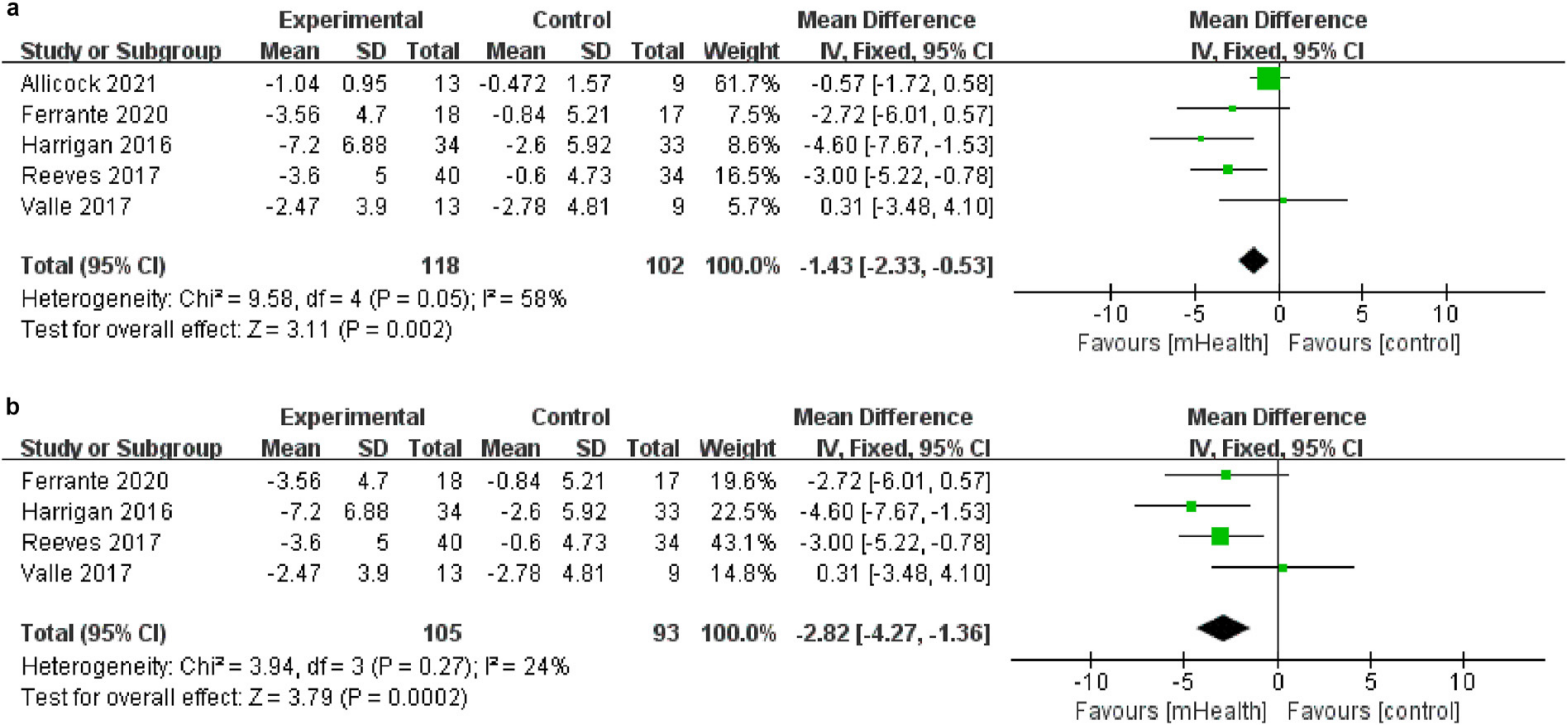
(a) Effects of mHealth diet interventions on waist circumference. (b) Sensitivity analysis of mHealth diet interventions on waist circumference.

Five studies,^19^^,^^20^^,^^31^^,^^36^^,^^37^ involving 220 patients, evaluated waist circumference; these studies indicated significant effects of mHealth diet interventions on this outcome (MD ​= ​−1.43, 95% CI [−2.33, −0.53], *P* ​< ​0.01; Fig. 8a). Waist circumference was measured by using a measuring tape. There was heterogeneity among studies (*I*^2^ ​= ​58%, *P* ​= ​0.05); thus, the random-effects model was used. To reduce this significant heterogeneity, a sensitivity analysis was conducted by sequentially excluding individual papers. After one study^36^ was removed, no significant heterogeneity was observed among the remaining five studies (*I*^*2*^ ​= ​24%, *P* ​= ​0.27; Fig. 8b). The fixed-effects model showed that mHealth diet interventions significantly reduced the waist circumference of cancer survivors (MD ​= ​−2.82, 95% CI [−4.27, −1.36], *P* ​< ​0.01; Fig. 8b).

#### 3.4.7 Effects of mHealth diet interventions on hip circumference

**Fig. 9:**
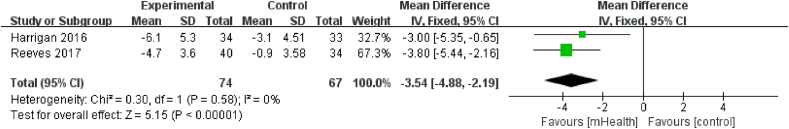
Effects of mHealth diet interventions on hip circumference.

Two studies,^19^^,^^31^ involving 141 patients, evaluated hip circumference. Hip circumference was measured by using tape. A fixed-effects model, the analysis revealed significant effects of mHealth diet interventions on hip circumference (MD ​= ​−3.54, 95% CI [–4.88, –2.19], *P* < 0.01) without substantial heterogeneity (*I*^*2*^ ​= ​0%, *P* ​= ​0.58; Fig. 9).

#### 3.4.8 Effects of mHealth diet interventions on QoL

**Fig. 10:**
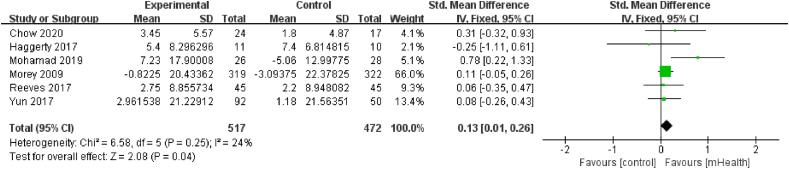
Effects of mHealth diet interventions on quality of life.

Six articles,^22^^,^^31^^,^^33^^,^^38^^,^^39^^,^^41^ involving 989 patients, evaluated QoL. The scales used in these trials included the Quality of Life in Adult Cancer Survivors Scale (QLACS), Short Form (SF)-36 Version 2 Health Survey, European Organization for Research and Treatment of Cancer Quality of Life Questionnaire (EORTC QLQ-C30), SF-36, Patient-Reported Outcomes Measurement Information System (PROMIS Global 10), and 12-Item Short Form Health Survey (SF-12). There was no heterogeneity among studies in this analysis (*I*^*2*^ ​= ​24%, *P* ​= ​0.25). MHealth diet interventions had a positive effect on QoL compared with control conditions (SMD ​= ​0.13, 95% CI [0.01, 0.26], *P* ​= ​0.04; Fig. 10).

### 3.5 Publication bias

**Fig. 11:**
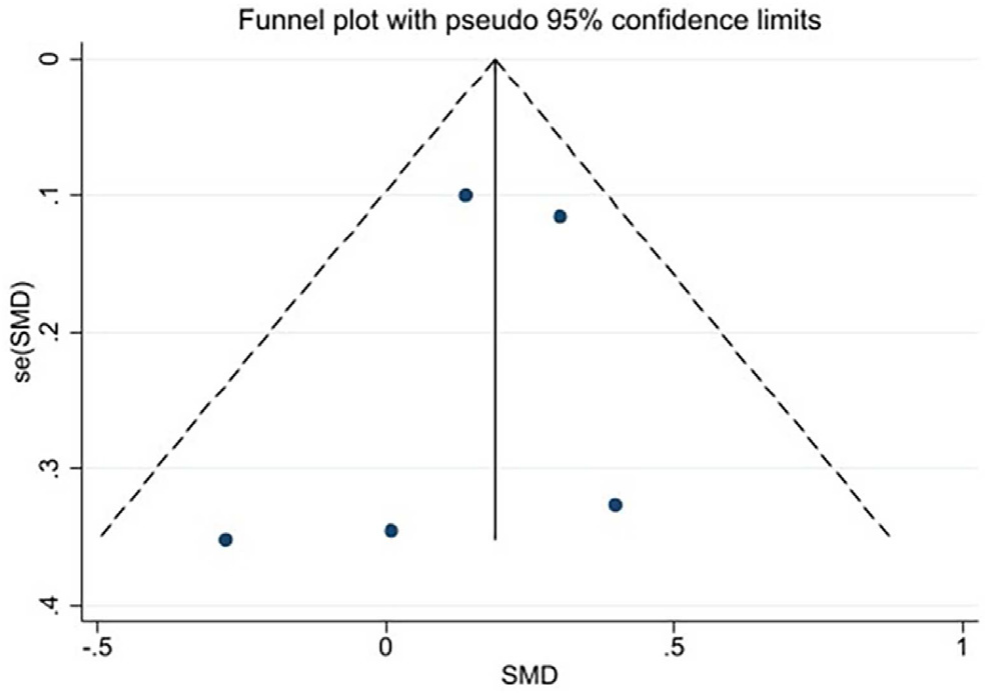
The funnel plot of fruit and vegetable intake.

**Fig. 12:**
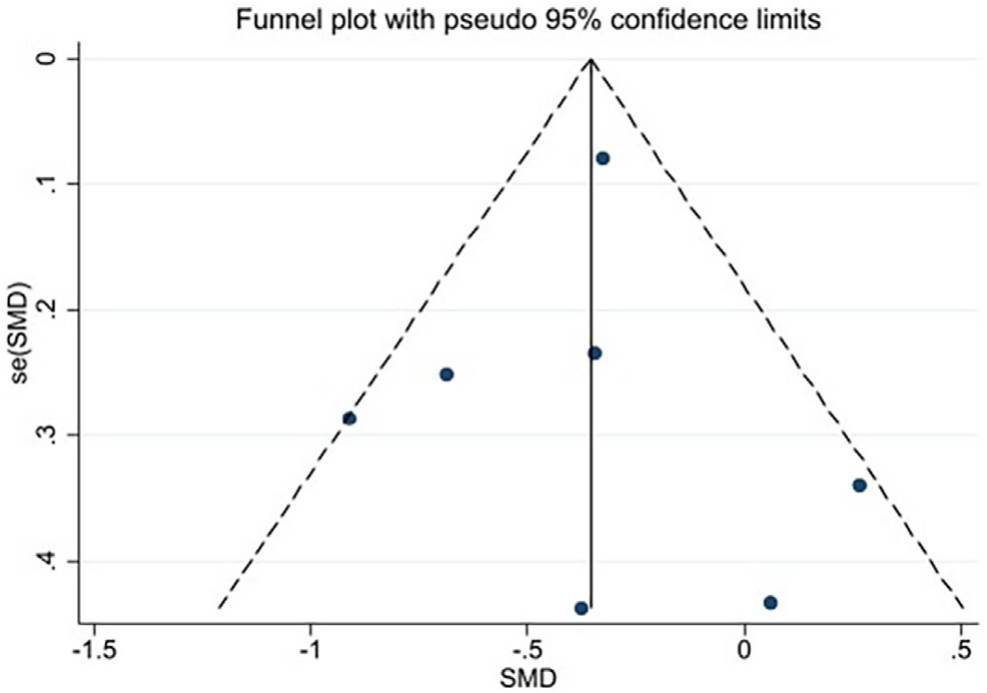
The funnel plot of weight change.

**Fig. 13:**
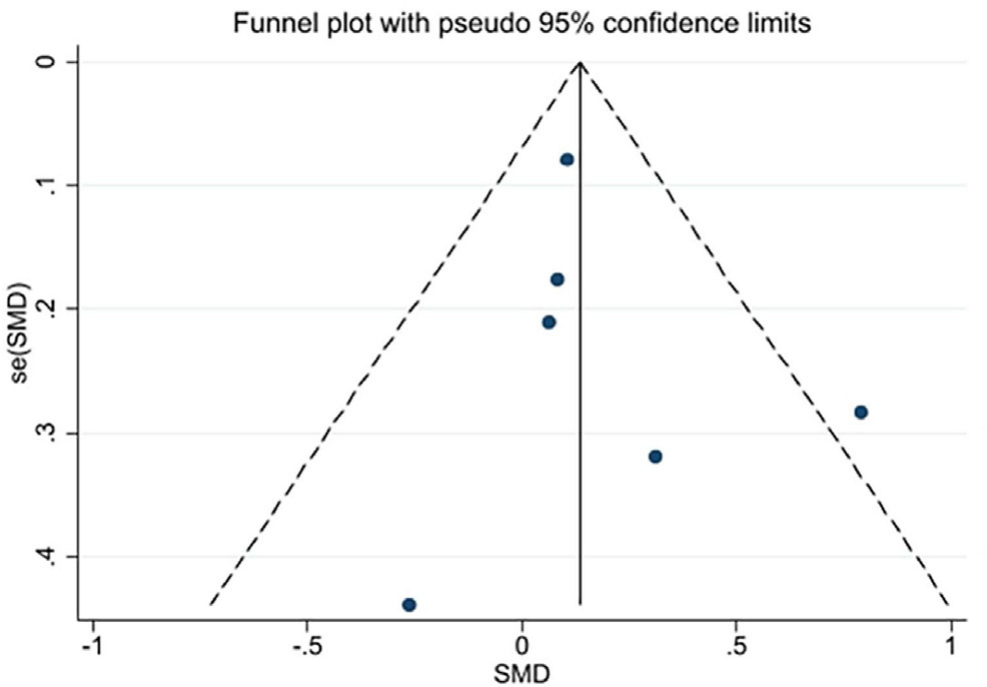
The funnel plot of quality of life.

Visual inspection of the funnel plot and the Egger test (*P* = 0.564, *P* = 0.948, *P* = 0.592 ) identified no publication bias (Fig. 11, Fig. 12, Fig. 13). Since the number of included studies for other outcomes was too small (*n* < 5), funnel plots were not constructed.

## 4 Discussion

To the best of our knowledge, this is the first systematic review and meta-analysis of the effects of mHealth diet interventions on cancer survivors. The results indicated that there was significant improvement in vegetable and fruit intake and QoL, reductions in fat intake, weight, waist circumference, and hip circumference, and no significant changes in energy intake or whole grain intake in cancer survivors after mHealth diet interventions.

The results show that the mHealth diet intervention increased fruit and vegetable intake and reduced fat intake in cancer survivors. Nutrition guidelines emphasize the importance of personalized nutrition counseling to help cancer survivors develop healthy eating habits including increased fruit and vegetable intake and reduced fat intake.^11^^,^^43^ Our findings were consistent with the recommendations of these guidelines. A variety of studies have tested the effects of nutritional interventions, including nutrition clinics, nutrition education, and printed materials on developing healthy eating habits in cancer survivors, but these studies have observed only small benefits.^44^^,^^45^ Our study found that mHealth diet interventions had an obvious effect on cancer survivors' healthy eating habits. The superiority of mHealth diet intervention may be due to mHealth enabling long-term and individualized intervention. Patients could set specific, achievable goals and receive relevant information, such as individually tailored dietary advice, individual guidance to achieve diet goals, and tailored progress reports. The mHealth diet intervention also increased cancer survivors’ interactions with the medical staff. Therefore, cancer survivors were more likely to change their diet behaviors and develop healthy eating habits. mHealth interventions had no effect on energy intake in cancer survivors. This finding was consistent with the results of other studies.^46^^,^^47^ Further well-designed RCTs with larger sample sizes are necessary to evaluate the effects of mHealth diet interventions on energy intake in cancer survivors.

MHealth diet interventions had no effect on whole grain intake in cancer survivors. The two studies included in this analysis both concluded that mHealth diet interventions could improve whole grain intake in cancer survivors, but after merging these studies in our meta-analysis, we obtained the opposite result. This was due to the use of a random-effects model to account for the high heterogeneity in data due to the far better outcomes of Van Blarigan's study compared to Parsons's. However, we could obtain a positive result by using a fixed-effects model. In our opinion, mHealth diet interventions likely benefit whole grain intake in cancer survivors, since the two studies were well designed. However, further prospective studies are needed to confirm this finding.

The results of our meta-analysis suggested that mHealth diet interventions can significantly reduce weight, waist circumference, and hip circumference. This may be due to the individualized health diet intervention. At present, targeted diet interventions focus on either weight loss or encouraging weight maintenance in cancer survivors.^48^ The most common topics in mHealth nutritional interventions were related to increasing fruit and vegetable intake and whole grain intake.^49^ Increased fruit and vegetable intake were similarly associated with reduced weight.^50^^,^^51^ Diet intervention combined with oral liquid nutritional supplements has an effect on encouraging weight maintenance in cancer survivors who experience weight loss or even cachexia.^52^^,^^53^ MHealth diet interventions can help cancer survivors change their diet behaviors and develop healthy eating habits. Therefore, mHealth diet intervention merits clinical use and should be regarded as a crucial component of comprehensive nutrition support for cancer survivors who experience weight loss or even cachexia. Reductions in waist circumference and hip circumference are related to weight loss.^54^^,^^55^ In addition, some studies have suggested that nutritional interventions should be combined with exercise to improve weight change in cancer survivors.^42^^,^^48^ However, in this study, we explored only the effects of mHealth diet interventions on cancer survivors.

In addition, mHealth diet interventions improved QoL in cancer survivors. There are possible reasons for this increase QoL. First, mHealth diet interventions increased interactions between health professionals and cancer survivors. Perceived increases in contact with health care providers is an important determinant of QoL of cancer survivors.^56^ Second, cancer survivors must be aware in many situations to adhere to a healthy diet.^57^ Changes in diet behaviors have a positive effect on nutritional intake, which may improve the body composition and symptom experience of cancer survivors.^58^^,^^59^ A study by Jones revealed that cancer survivors are particularly concerned with their nutritional status.^60^ Finally, diet significantly affects the mood of survivors. Healthy diet behaviors lead to more positive emotions.^61^ Therefore, nutrition delivered via mHealth interventions is well accepted by cancer survivors allowing them to improve their QoL.

Based on the findings of this study, we recommend using individualized nutritional interventions delivered via phone calls, text messages, and mobile apps could improve the fruit and vegetable intake, and QoL, of cancer survivors. The most recommended frequency and length of mHealth diet interventions were weekly and 6 months, respectively. MHealth interventions that combine nutrition with exercise are expected to benefit weight change in cancer survivors. Cancer survivors, academicians, and health professionals could benefit from the results of this study. Health professionals could obtain the methods for mHealth diet interventions. MHealth diet interventions can provide remote access to individual nutritional interventions to cancer survivors that change their diet behaviors. This research can guide future researchers to design interventions that benefit cancer survivors with more economical methods and improved outcomes.

### 4.1 Limitations

There were several limitations of this meta-analysis. First, all included studies were published in English. Due to language restrictions, some articles may have been omitted. Second, this meta-analysis mainly focused on the effectiveness of mHealth interventions on diet intake; thus, one limitation of this meta-analysis is that other behaviors that may also contribute to weight change (eg, activity) were not included. Third, this study was not registered in the International Prospective Register of Systematic Reviews (PROSPERO). Fourth, we did not perform subgroup analysis and thus could not determine the most effective mHealth diet interventions. These limitations mean that our results should be interpreted with caution.

## 5 Conclusions

Our study provides preliminary data for the future development and application of mHealth diet interventions in cancer survivors. This systematic review and meta-analysis showed that mHealth diet interventions improve fruit and vegetable intake, and QoL and significantly reduce fat intake, weight, waist circumference, and hip circumference in cancer survivors. However, there was insufficient evidence regarding the effects of these interventions on energy intake or whole grain intake. This study raises awareness of mHealth diet interventions and encourages health professionals to implement them to improve the diet of cancer survivors.

## CRediT author statement

**Yabo GONG**: Conceptualization, Methodology, Writing – Original Draft. **Xiaohan JIANG**: Software, Validation, Writing – Review and Editing. **Xi-jie CHEN**: Resources, Data Curation, Visualization. **Shi CHEN**: Formal Analysis, Investigation. **Yue WEN**: Methodology. **Xiuhong YUAN**: Data Curation, Visualization. **Jiamin CHEN**: Supervision. **Junsheng PENG**: Writing – Review and Editing, Project Administration, Funding Acquisition. All authors had full access to all the data in the study, and the corresponding author had final responsibility for the decision to submit for publication. The corresponding author attests that all listed authors meet authorship criteria and that no others meeting the criteria have been omitted.

## Declaration of competing interest

The authors declare no conflict of interest.

## Funding

Research Fund of the Sixth Affiliated Hospital of 10.13039/501100002402Sun Yat-sen University (Grant No. P20200217202309876). The funders had no role in considering the study design or in the collection, analysis. Interpretation of data, writing of the report, or decision to submit the article for publication. They do not receive compensation from pharmaceutical companies or other than those listed above, and the authors declare no conflict of interest.

## Ethics statement

Not required.

## Data availability statement

Data availability is not applicable to this article as no new data were created or analyzed in this study.
