# Supplementary material for: Effectiveness of mHealth diet interventions in cancer survivors: A systematic review and meta-analysis of randomized controlled trials
Source: Asia Pac J Oncol Nurs. 2023 Feb 14;10(3):100196. doi: 10.1016/j.apjon.2023.100196 (PMC10140457; doi:10.1016/j.apjon.2023.100196)
Supplement: Multimedia component 1 [file mmc1.docx]

**Supplementary Material**

**Table 1 Search Strategies**

| **Database** | **Searching time** | **Search strategy** | **Results** |
| --- | --- | --- | --- |
| Pubmed | 25 September 2022 | #1 ((((((((neoplasms[MeSH Terms]) OR (neoplasms[Title/Abstract])) OR (cancer[Title/Abstract])) OR (oncology[Title/Abstract])) OR (tumour[Title/Abstract])) OR (tumor[Title/Abstract])) OR (malignancy[Title/Abstract])) OR (carcinoma[Title/Abstract])) OR (leukemia[Title/Abstract]) | 475787 |
|  |  | #2 ((((((((((((((((((((((((((((((((telemedicine[MeSH Terms]) OR (telemedicine[Title/Abstract])) OR (mHealth[Title/Abstract])) OR (mobile health[Title/Abstract])) OR (telehealth[Title/Abstract])) OR (eHealth[Title/Abstract])) OR (mobile applications[MeSH Terms])) OR (mobile applications[Title/Abstract])) OR (mobile application[Title/Abstract])) OR (mobile apps[Title/Abstract])) OR (mobile app[Title/Abstract])) OR (portable electronic app[Title/Abstract])) OR (portable electronic application[Title/Abstract])) OR (portable software app[Title/Abstract])) OR (portable software app[Title/Abstract])) OR (smartphone[MeSH Terms])) OR (smartphone[Title/Abstract])) OR (smartphones[Title/Abstract])) OR (smart phone[Title/Abstract])) OR (texting[Title/Abstract])) OR (short message service[Title/Abstract])) OR (text messaging[MeSH Terms])) OR (text messaging[Title/Abstract])) OR (text message[Title/Abstract])) OR (cell phone[MeSH Terms])) OR (cell phone[Title/Abstract])) OR (cellular phone[Title/Abstract])) OR (cellular telephone[Title/Abstract])) OR (portable cellular phone[Title/Abstract])) OR (transportable cellular phone[Title/Abstract])) OR (mobile phone[Title/Abstract])) OR (mobile telephone[Title/Abstract])) OR (online intervention[Title/Abstract]) | 105395 |
|  |  | #3 ((((((((((diet[MeSH Terms]) OR (diet[Title/Abstract])) OR (diets[Title/Abstract])) OR (diet pattern[Title/Abstract])) OR (diet patterns[Title/Abstract])) OR (diet quality[Title/Abstract])) OR (dietary quality[Title/Abstract])) OR (eating pattern[Title/Abstract])) OR (eating patterns[Title/Abstract])) OR (eating behaviours[Title/Abstract])) OR (eating behaviour[Title/Abstract]) | 583697 |
|  |  | #4 #1 AND #2 AND #3 | 146 |
| EMBASE | 25 September 2022 | #1 'neoplasm'/exp OR neoplasm:ab,ti OR cancer:ab,ti OR 'oncology'/exp OR oncology:ab,ti OR tumour:ab,ti OR tumor:ab,ti OR malignancy:ab,ti OR carcinoma:ab,ti OR leukemia:ab,ti | 6704312 |
|  |  | #2 'telemedicine'/exp OR telemedicine:ab,ti OR mhealth:ab,ti OR 'mobile health'/exp OR 'mobile health':ab,ti OR 'telehealth'/exp OR telehealth:ab,ti OR ehealth:ab,ti OR 'mobile application'/exp OR 'mobile application':ab,ti OR 'mobile apps':ab,ti OR 'mobile app':ab,ti OR 'portable electronic app':ab,ti OR 'portable electronic application':ab,ti OR 'portable software app':ab,ti OR 'smartphone'/exp OR smartphone:ab,ti OR smartphones:ab,ti OR 'smart phone':ab,ti OR texting:ab,ti OR 'short message service'/exp OR 'short message service':ab,ti OR 'text messaging'/exp OR 'text messaging':ab,ti OR 'text message'/exp OR 'text message':ab,ti OR 'cellular phone':ab,ti OR 'cellular telephone':ab,ti OR 'cell phone':ab,ti OR 'portable cellular phone':ab,ti OR 'transportable cellular phone':ab,ti OR 'mobile phone':ab,ti OR 'mobile telephone':ab,ti OR 'online intervention':ab,ti | 144060 |
|  |  | #3 'diet'/exp OR diet:ab,ti OR 'diets'/exp OR diets:ab,ti OR 'dietary pattern'/exp OR 'diet pattern':ab,ti OR 'diet patterns':ab,ti OR 'diet quality'/exp OR 'diet quality':ab,ti OR 'dietary quality'/exp OR 'dietary quality':ab,ti OR 'eating pattern'/exp OR 'eating pattern':ab,ti OR 'eating patterns':ab,ti OR 'eating behaviours':ab,ti OR 'eating behaviour':ab,ti | 681790 |
|  |  | #4 #1 AND #2 AND #3 | 274 |
| Web of Science | 25 September 2022 | #1 (((((((TS=(neoplasms)) OR TS=(cancer)) OR TS=(oncology)) OR TS=(tumour)) OR TS=(tumor)) OR TS=(malignancy)) OR TS=(carcinoma)) OR TS=(leukemia) All Databases | 8313258 |
|  |  | #2 (((((((((((((((((((((((((((TS=(telemedicine)) OR TS=(mHealth)) OR TS=(mobile health)) OR TS=(telehealth)) OR TS=(eHealth)) OR TS=(mobile applications)) OR TS=(mobile application)) OR TS=(mobile apps)) OR TS=(mobile app)) OR TS=(portable electronic app)) OR TS=(portable electronic application)) OR TS=(portable software app)) OR TS=(portable software app)) OR TS=(smartphone)) OR TS=(smartphones)) OR TS=(smart phone)) OR TS=(texting)) OR TS=(short message service)) OR TS=(text messaging)) OR TS=(text message)) OR TS=(cell phone)) OR TS=(cellular phone)) OR TS=(cellular telephone)) OR TS=(portable cellular phone)) OR TS=(transportable cellular phone)) OR TS=(mobile phone)) OR TS=(mobile telephone)) OR TS=(online intervention) All Databases | 2125237 |
|  |  | #3 (((((((((TS=(diet)) OR TS=(diets)) OR TS=(diet pattern)) OR TS=(diet patterns)) OR TS=(diet quality)) OR TS=(dietary quality)) OR TS=(eating pattern)) OR TS=(eating patterns)) OR TS=(eating behaviours)) OR TS=(eating behaviour) All Databases | 1316020 |
|  |  | #5 #1 AND #2 AND #3 | 654 |
| Cochrane | 25 September 2022 | #1 MeSH descriptor: [Neoplasms] explode all trees | 89835 |
|  |  | #2 (neoplasms):ti,ab,kw OR (cancer):ti,ab,kw OR (oncology):ti,ab,kw OR (tumour):ti,ab,kw OR (tumor):ti,ab,kw | 230188 |
|  |  | #3 (malignancy):ti,ab,kw OR (carcinoma):ti,ab,kw OR (leukemia):ti,ab,kw | 65841 |
|  |  | #4 #1 OR #2 OR #3 | 255574 |
|  |  | #5 MeSH descriptor: [Telemedicine] explode all trees | 3306 |
|  |  | #6 MeSH descriptor: [Mobile Applications] explode all trees | 1127 |
|  |  | #7 MeSH descriptor: [Smartphone] explode all trees | 683 |
|  |  | #8 MeSH descriptor: [Text Messaging] explode all trees | 1151 |
|  |  | #9 MeSH descriptor: [Cell Phone] explode all trees | 2381 |
|  |  | #10 (telemedicine):ti,ab,kw OR (mHealth):ti,ab,kw OR (Mobile Health):ti,ab,kw OR (Telehealth):ti,ab,kw OR (eHealth):ti,ab,kw | 19941 |
|  |  | #11 (Portable Electronic Application):ti,ab,kw OR (Portable Software App):ti,ab,kw OR (Portable Software App):ti,ab,kw OR (smartphone):ti,ab,kw OR (smartphones):ti,ab,kw | 6073 |
|  |  | #12 (Smart Phone):ti,ab,kw OR (Texting):ti,ab,kw OR (Short Message Service):ti,ab,kw OR (Text Messaging):ti,ab,kw OR (Text Message):ti,ab,kw | 14917 |
|  |  | #13 (Cell Phone):ti,ab,kw OR (Cellular Phone):ti,ab,kw OR (Cellular Telephone):ti,ab,kw OR (Portable Cellular Phone):ti,ab,kw OR (Transportable Cellular Phone):ti,ab,kw | 2300 |
|  |  | #14 (Mobile Phone):ti,ab,kw OR (Mobile Telephone):ti,ab,kw OR (online intervention):ti,ab,kw | 17633 |
|  |  | #15 #5 OR #6 OR #7 OR #8 OR #9 OR #10 OR #11 OR #12 OR #13 OR #14 | 48010 |
|  |  | #16 MeSH descriptor: [Diet] explode all trees | 20453 |
|  |  | #17 (diet):ti,ab,kw OR (diets):ti,ab,kw OR (diet pattern):ti,ab,kw OR (diet patterns):ti,ab,kw OR (diet quality):ti,ab,kw | 72030 |
|  |  | #18 (dietary quality):ti,ab,kw OR (eating pattern):ti,ab,kw OR (eating patterns):ti,ab,kw OR (eating behaviours):ti,ab,kw OR (eating behaviour):ti,ab,kw | 15517 |
|  |  | #19 #16 OR #17 OR #18 | 84426 |
|  |  | #20 MeSH descriptor: [Randomized Controlled Trial] explode all trees | 118 |
|  |  | #21 (RCT):ti,ab,kw OR (randomized controlled trial):ti,ab,kw OR (randomised controlled trial):ti,ab,kw OR (randomized control trial):ti,ab,kw OR (randomized controlled trial):ti,ab,kw | 747067 |
|  |  | #22 (controlled trial):ti,ab,kw | 818097 |
|  |  | #23 #20 OR #21 OR #22 | 823685 |
|  |  | #24 #4 AND #15 AND #19 AND #22 | 270 |
| Scopus | 25 September 2022 | ( TITLE-ABS-KEY ( neoplasms  OR  cancer  OR  oncology  OR  tumour  OR  tumor  OR  malignancy  OR  carcinoma  OR  leukemia )  AND  TITLE-ABS-KEY ( telemedicine  OR  mhealth  OR  "mobile health"  OR  telehealth  OR  ehealth  OR  "mobile applications"  OR  "mobile application"  OR  "mobile apps"  OR  "mobile app"  OR  "portable electronic app"  OR  "portable electronic application"  OR  "portable software app"  OR  "portable software app"  OR  smartphone  OR  smartphones  OR  "smart phone"  OR  texting  OR  "short message service"  OR  "text messaging"  OR  "text message"  OR  "cell phone"  OR  "cellular phone"  OR  "cellular telephone"  OR  "portable cellular phone"  OR  "transportable cellular phone"  OR  "mobile phone"  OR  "mobile telephone"  OR  "online intervention" )  AND  TITLE-ABS-KEY ( diet  OR  diets  OR  "diet pattern"  OR  "diet patterns"  OR  "diet quality"  OR  "dietary quality"  OR  "eating pattern"  OR  "eating patterns"  OR  "eating behaviours"  OR  "eating behaviour" )  AND  TITLE-ABS-KEY ( rct  OR  "randomized controlled trial"  OR  "randomised controlled trial"  OR  "randomized control trial"  OR  "randomized controlled trial" ) ) | 88 |
| ProQuest | 25 September 2022 | #1 title( "neoplasms" ) OR title("cancer") OR title("oncology" ) OR title( "tumour") OR title("tumor" ) OR title("malignancy" ) OR title( "carcinoma" ) OR title( "leukemia" ) | 3710554 |
|  |  | #2 abstract("neoplasms" ) OR abstract("cancer") OR abstract("oncology" ) OR abstract( "tumour") OR abstract("tumor" ) OR abstract("malignancy" ) OR abstract( "carcinoma" ) OR abstract("leukemia" ) | 5094535 |
|  |  | #3 #1 OR #2 | 6291555 |
|  |  | #4 title("telemedicine" ) OR title("mhealth" ) OR title("mobile health" ) OR title("telehealth" ) OR title("ehealth" ) OR title( "mobile applications" ) OR title("mobile application") OR title("mobile apps" ) OR title("mobile app" ) OR title("portable electronic app" ) | 125244 |
|  |  | #5 title("portable electronic application") OR title( "portable software app") OR title("portable software app") OR title( "smartphone" ) OR title( "smartphones" ) OR title( "smart phone") OR title("texting" ) OR title("short message service" ) OR title( "text messaging" ) OR title("text message" ) | 126128 |
|  |  | #6 title("cell phone" ) OR title("cellular phone" ) OR title( "cellular telephone" ) OR title("portable cellular phone") OR title( "transportable cellular phone" ) OR title( "mobile phone" ) OR title( "mobile telephone" ) OR title("online intervention") | 68034 |
|  |  | #7 abstract("telemedicine" ) OR abstract("mhealth" ) OR abstract("mobile health" ) OR abstract("telehealth" ) OR abstract("ehealth" ) OR abstract("mobile applications" ) OR abstract("mobile application") OR abstract("mobile apps" ) OR abstract("mobile app") OR abstract("portable electronic app") | 101956 |
|  |  | #8 abstract("portable electronic application") OR abstract( "portable software app") OR abstract("portable software app") OR abstract("smartphone" ) OR abstract("smartphones" ) OR abstract("smart phone") OR abstract("texting" ) OR abstract("short message service") OR abstract( "text messaging" ) OR abstract("text message" ) | 120789 |
|  |  | #9 abstract("cell phone" ) OR abstract("cellular phone" ) OR abstract("cellular telephone") OR abstract("portable cellular phone") OR abstract("transportable cellular phone") OR abstract("mobile phone" ) OR abstract( "mobile telephone" ) OR abstract("online intervention") | 75442 |
|  |  | #10 #4 OR #5 OR #6 OR #7 OR #8 OR #9 | 739081 |
|  |  | #11 title(”diet“) OR title("diets" ) OR title( "diet pattern" ) OR title( "diet patterns") OR title("diet quality" ) OR title( "dietary quality") OR title("eating pattern" ) OR title("eating patterns" ) OR title("eating behaviours") OR title("eating behaviour") | 311788 |
|  |  | #12 abstract(”diet“) OR abstract("diets" ) OR abstract("diet pattern") OR abstract("diet patterns") OR abstract("diet quality") OR abstract( "dietary quality") OR abstract("eating pattern" ) OR abstract("eating patterns" ) OR abstract("eating behaviours") OR abstract("eating behaviour") | 933611 |
|  |  | #13 #11 OR #12 | 1058191 |
|  |  | #14 title(“RCT") OR title( "randomized controlled trial") OR title( "randomised controlled trial" ) OR title("randomized control trial" ) OR title( "randomized controlled trial") OR abstract(“RCT") OR abstract( "randomized controlled trial") OR abstract( "randomised controlled trial" ) OR abstract("randomized control trial" ) OR abstract( "randomized controlled trial") | 280255 |
|  |  | #15 #3 AND #10 AND #13 AND #14 | 271 |
| CNKI | 26 September 2022 | #1篇关摘 (癌症 + 癌 + 肿瘤 + 恶性肿瘤) | 2068400 |
|  |  | #2篇关摘 (移动医疗 + 远程医疗 + 移动应用 + 电话 + 手机 + 短信 + 便携式软件应用 + 在线干预 + 线上) | 813300 |
|  |  | #3篇关摘 (饮食 + 膳食 + 饮食行为 + 饮食模式 + 饮食质量) | 392500 |
|  |  | #4篇关摘 (随机 + 随机对照试验 + 随机对照 + RCT) | 2462000 |
|  |  | #5 #1 AND #2 AND #3 AND #4 | 91 |
| WanFang | 26 September 2022 | #1 主题 (癌症 OR 癌 OR 肿瘤 OR 恶性肿瘤) | 2607055 |
|  |  | #2 主题 (移动医疗 OR 远程医疗 OR 移动应用 OR 电话 OR 手机 OR 短信 OR 便携式软件应用 OR 在线干预 OR 线上) | 3542252 |
|  |  | #3 主题 (饮食 OR 膳食 OR 饮食行为 OR 饮食模式 OR 饮食质量) | 455343 |
|  |  | #4 主题（随机 OR 随机对照试验 OR 随机对照 OR RCT) | 3145495 |
|  |  | #5 #1 AND #2 AND #3 AND #4 | 802 |
| SinoMed | 26 September 2022 | #1 "癌症"[常用字段:智能] OR "癌"[常用字段:智能] OR "肿瘤"[常用字段:智能] OR "恶性肿瘤"[常用字段:智能] | 1988303 |
|  |  | #2 "移动医疗"[常用字段:智能] OR "移动医疗"[常用字段:智能] OR "移动应用"[常用字段:智能] OR "电话"[常用字段:智能] OR "手机"[常用字段:智能] OR "短信"[常用字段:智能] OR "便携式软件应用"[常用字段:智能] OR "在线干预"[常用字段:智能] OR "线上"[常用字段:智能] | 45394 |
|  |  | #3 "饮食"[常用字段:智能] OR "膳食"[常用字段:智能] OR "饮食行为"[常用字段:智能] OR "饮食模式"[常用字段:智能] OR "饮食质量"[常用字段:智能] | 174098 |
|  |  | #4 "随机"[常用字段:智能] OR "随机对照试验"[常用字段:智能] OR "随机对照"[常用字段:智能] OR "RCT"[常用字段:智能] | 1820685 |
|  |  | #5 #1 AND #2 AND #3 AND #4 | 52 |
